# Differential Myopathic and Transcriptomic Changes in Soleus and Gastrocnemius Muscles in a Novel Chronic Hindlimb Ischemia Rat Model Induced by Endovascular Catheter Occlusion

**DOI:** 10.1111/apha.70278

**Published:** 2026-07-09

**Authors:** Oliver Kitzerow, Zhiqiu Xia, Samuel Gillman, Iraklis I. Pipinos, Han‐Jun Wang

**Affiliations:** ^1^ Department of Genetics, Cell Biology, and Anatomy University of Nebraska Medical Center Omaha Nebraska USA; ^2^ Department of Anesthesiology University of Nebraska Medical Center Omaha Nebraska USA; ^3^ Department of Medical Education Creighton University Omaha Nebraska USA; ^4^ Department of Surgery University of Nebraska Medical Center Omaha Nebraska USA

## Abstract

Peripheral artery disease (PAD) is a progressive atherothrombotic disorder affecting more than 230 million people worldwide. Conventional animal models of chronic hindlimb ischemia (HLI) are highly invasive, technically challenging, fail to account for anatomical variation, and may not accurately recapitulate progressive PAD pathophysiology. To address these limitations, we developed a novel chronic HLI model using an ilio‐femoral endovascular catheter occlusion (IFCO) approach. We hypothesized that IFCO would chronically reduce hindlimb blood flow, induce PAD‐associated myopathy, and overcome limitations of conventional techniques. Laser Doppler perfusion imaging demonstrated that IFCO reduced resting hindlimb perfusion by 70%, with significant reductions persisting for up to 28 days. Histological analysis of the soleus revealed significantly increased fibrosis (4.99% in sham vs. 13.19% in IFCO) and reduced myofiber cross‐sectional area (3072.21 μm^2^ in sham vs. 1556.27 μm^2^ in IFCO), whereas no significant differences were observed in the gastrocnemius. In contrast, IFCO increased the proportion of centralized nuclei from near 0% in sham muscles to 21.55% in the gastrocnemius and 32.65% in the soleus. RNA sequencing corroborated these findings and demonstrated that the ischemic soleus transcriptome exhibited enhanced fibrotic, pro‐angiogenic, and myoblast fusion‐associated signaling consistent with histological evidence of myopathy. These findings demonstrate that IFCO produces sustained hindlimb ischemia and PAD‐associated skeletal muscle remodeling, with distinct responses between muscle types. This model may facilitate studies of PAD‐associated myopathy and support the development of targeted therapeutic interventions.

## 1 Introduction

Peripheral arterial disease (PAD) is a progressive atherothrombotic disorder and one of the most prevalent cardiovascular diseases (CVDs), impacting > 200 million people worldwide [1, 2, 3]. PAD is associated with increased morbidity and increased risk of complications such as leg amputation and death [2, 4, 5]. The most common manifestation of PAD is intermittent claudication (IC) [6, 7]. Claudicating patients describe their symptoms as tightness, cramping, and deep aching in the muscles of the calf, thigh, or buttock. These symptoms occur during exercise and are relieved by resting, thus severely limiting physical activity and decreasing quality of life (QOL) [8, 9, 10]. Current medical management is mainly centered around reducing the risk of cardiovascular events [11]. In its most severe form, PAD is referred to as critical limb‐threatening ischemia (CLTI). Common treatments for CLTI include surgical revascularization with stents or bypasses [12, 13]. However, up to 30% of patients with CLTI are ineligible to receive revascularization procedures due to unfavorable anatomy, lack of suitable conduit, or prohibitive comorbidities and frailty, and when performed, these procedures have poor long‐term durability [12, 13, 14, 15, 16, 17]. Moreover, revascularization for claudication has not been shown to result in durable improvements in quality of life (QOL) [18]. Randomized trials indicate that while revascularization can provide short‐term improvements in walking distance and QOL, these benefits often diminish over time, and long‐term QOL is not consistently superior to medical therapy alone [19]. Thus, there is a pressing need for the development of novel medical therapies for PAD. Despite promising pre‐clinical results for novel therapies like stem cells and angiogenic growth factors, the translation of benefits to large‐scale human studies has not been regularly reproduced [20, 21, 22, 23, 24, 25, 26]. Possible explanations include the enrollment of patients who had already undergone standard therapies and may therefore present with recalcitrant or difficult‐to‐treat symptoms, small sample sizes, the absence of validation studies, and limitations inherent to the pre‐clinical animal models used.

Animal studies are essential for advancing our understanding of PAD pathophysiology and treatment strategies. An appropriate PAD model should effectively reproduce key clinical and pathological characteristics, such as reduced muscle fibrosis and atrophy, and reduced cross‐sectional area of muscle fibers. Animal HLI models are also far more cost‐ and time‐efficient than human clinical studies when testing new therapies and optimizing their parameters [27]. The ideal animal model would: “(1) incorporate relevant human risk factors, (2) be low cost, (3) be easy to establish and maintain, and (4) support detailed study of cellular and molecular mechanisms and treatment interventions” [28]. However, there is considerable variability in animal HLI models, including differences in surgical procedures, animal strain and age, and a lack of standardized functional outcomes [28]. Historically, the most commonly used techniques to induce animal HLI involve surgical ligation of the femoral artery and its branches [27, 28, 29, 30, 31, 32]. However, ligation techniques do not produce chronic reductions in the resting blood flow [33, 34] were the first to describe a chronic HLI model involving ligation and complete excision of the femoral artery. In this procedure, the distal external iliac artery, inferior epigastric, profunda femoral, circumflex femoral, and superior epigastric arteries were ligated proximally, and the popliteal and saphenous arteries were ligated distally, followed by complete excision of the femoral artery. While such models may approximate PAD features, such as reduced exercise tolerance and hindlimb blood flow, they do not account for animal‐to‐animal anatomical variation, are highly invasive, and are technically demanding.

**FIGURE 1:**
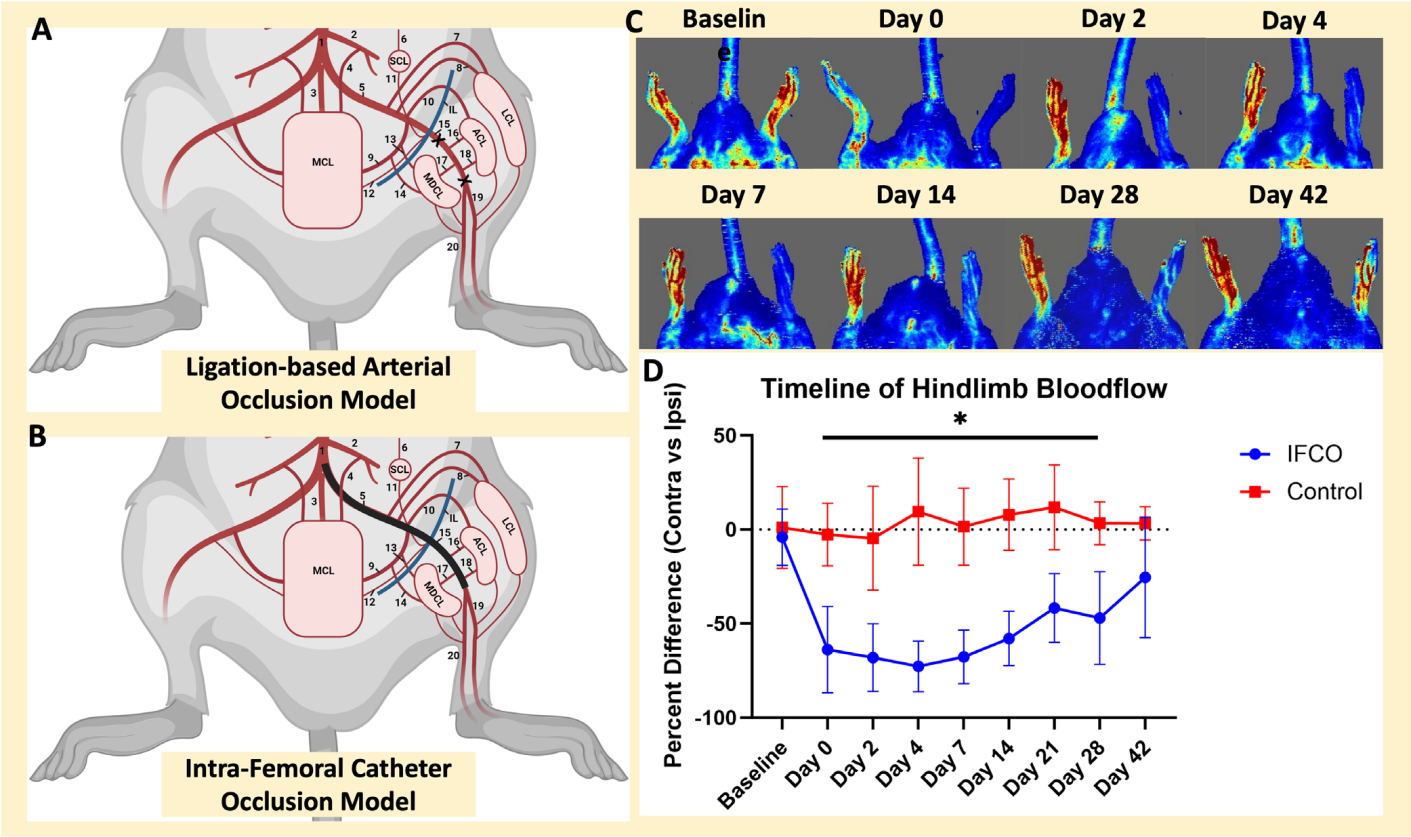
Ligation vs. IFCO arterial schematic of the rat hindlimb. (A) Traditional ligation and excision techniques (shown as X's) used to induce HLI do not encompass all perfusion territories. (B) IFCO extends from the distal iliofemoral artery to the aortic bifurcation, thus blocking and limiting collateral flow. (1) aorta, (2) lumbar artery, (3) median sacral artery, (4) iliolumbar artery, (5) common iliac artery, (6) inferior epigastric artery, (7) cranial gluteal artery, (8) caudal gluteal artery, (9) internal iliac artery, (10) iliofemoral artery, (11) caudal epigastric artery, (12) external pudendal artery, (13) deep femoral artery, (14) obturator artery, (15) femoral artery, (16) lateral circumflex femoral artery, (17) proximal caudal femoral artery, (18) superficial caudal epigastric artery, (19) popliteal artery, (20) saphenous artery. SCL, superior collateral zone; MCL, median collateral zone; MDCL, medial deep collateral zone; ACL, anterior collateral zone; LCL, lateral collateral zone. (C) Representative timeline images of LDPI starting at baseline up to 42 days post‐IFCO surgery. (D) Average differences between contra‐ and ipsilateral limb perfusion of IFCO and sham rats. Mean ± SD. *p* < 0.05 (*), *n = 6–14 per group*.

The present study describes a new rodent chronic HLI model using an intra‐arterial, catheter‐based approach, which was validated by assessing laser Doppler perfusion and myopathy associated with HLI. Our method enables consistent occlusion of collateral and anastomotic blood flow without complete dissection and excision of the femoral artery and its branches (Figure 1A,B). This approach is less invasive because it reduces the extent of femoral dissection and the risk of bleeding, thereby decreasing perioperative morbidity and mortality. Because it uses a single insertion site, the procedure requires less technical skill and can be completed more quickly. We hypothesized that endovascular occlusion of the iliofemoral artery using an indwelling catheter would produce sustained reductions in hindlimb perfusion, resulting in chronic ischemic skeletal muscle remodeling characteristic of PAD, including myofiber degeneration, fibrosis, and regenerative changes. This approach would overcome key limitations of conventional surgical HLI models by providing reproducible and persistent ischemia while minimizing surgical invasiveness. Accordingly, the objectives of this study were to determine whether ilio‐femoral catheter occlusion (IFCO) produces chronic hindlimb ischemia, to characterize the resulting histological and transcriptomic features of ischemic myopathy, and to evaluate its utility as a preclinical model of PAD.

## 2 Methods

### 2.1 Rats

All animal experimentation was approved by the Institutional Animal Care and Use Committee of the University of Nebraska Medical Center and performed in accordance with the National Institutes of Health's Guide for Use and Care of Laboratory Animals and in accordance with the ARRIVE guidelines [35, 36]. Rat experiments were performed on adult, male ~300–350 g Sprague–Dawley rats purchased from the Charles River Laboratories (Wilmington, MA, United States). Animals were housed on‐site and given a one‐week acclimation period before experimentation. Food and water were supplied ad libitum and were kept on a 12‐h light/dark cycle.

### 2.2 Intra‐Arterial Catheter HLI Model

Rats 12–16 weeks old were randomly selected to undergo sham or IFCO surgery. Anesthesia was induced with a mixture of oxygen and 2%–3% isoflurane. A left groin incision was made over the femoral triangle. The left femoral artery was exposed and carefully dissected free of the femoral vein and nerve. A ligature was placed around the distal end of the femoral artery proximal to the saphenous/popliteal bifurcation to prevent bleeding upon arteriotomy. Next, a temporary clamp was applied to the femoral artery just distal to the inguinal ligament to arrest forward flow. Prior to arteriotomy, lidocaine (~0.2 mL) was applied topically to facilitate vasodilation and catheter insertion. Next, an arteriotomy was performed proximal to the saphenous/popliteal bifurcation, and a customized catheter, 3.0 cm‐long sterile PE30 polyethylene tubing (010522 G, Braintree Scientific Inc.) with its lumen sealed by a sterile 3–0 Surgipro monofilament polypropylene (CP‐454, Covidien, USA) was carefully introduced and advanced. The clamp was removed, and the catheter was secured in place with a ligature. The skin was then sutured. The animals were observed in the procedure room until they recovered from the anesthesia. Buprenorphine SR (1.5 mg/kg) was subcutaneously injected for post‐procedure analgesia. Sham procedures replicated all steps except the arteriotomy and catheter insertion. There was no perioperative mortality in the sham or IFCO cohorts.

### 2.3 Laser Doppler Perfusion Imaging

Perfusion was measured noninvasively using laser Doppler perfusion imaging (LDPI, MoorLDI2, Moor Instruments). Ambient light and temperature were maintained at 37°C core temperature. Rats were placed supine on a warming table with a black surface. Isoflurane (2.0% induction and 1.5% maintenance doses plus oxygen; 1 L/min) was used to anesthetize the animals. Rodents were evaluated under the same experimental conditions and were scanned from the lower abdomen to the end of the toes. The region of interest (ROI) spanned from the proximal ankle to the toes, so hair was not removed until surgery. The LDPI system was mounted 26 cm above the animal's limbs. Rats were randomly selected for IFCO surgery (*n* = 12) or sham surgery (*n* = 6). Imaging was performed at the following time points for rats: Prior to surgery, day 0—immediately after surgery, days 2, 4, 7, 14, 21, 28, and 42 after surgery. Image analysis software (Laser Doppler Perfusion Measure, V3.08, Moor Instruments) was used to calculate the limb mean flux units, which represent a quantitative analysis of tissue perfusion on a scale of 0 to 1 000 perfusion units (PU). Identical regions of both the ipsilateral ischemic and contralateral non‐ischemic limbs were assessed for perfusion quantification. Limb perfusion was expressed as the ratio of the flux value of the ischemic limb relative to the value of the contralateral non‐ischemic limb.

### 2.4 Histology

#### 2.4.1 Myopathy

Ipsilateral triceps surae muscles were collected from rats four weeks post‐IFCO (*n* = 19) or sham (*n* = 11) surgery and fixed in methacarn (American MasterTech Scientific Inc.) for 24 h. The tissues were then washed in 70% ethanol and embedded in paraffin. Sections (5 μm‐thick) were taken halfway between the muscle's origins and the musculotendinous junction. Tissue sections were stained with Hematoxylin and Eosin (H&E), Masson trichrome (MT), rabbit anti‐cleaved Caspase‐3 (1:400; Cell Signaling Technology, Antibody #9661), and examined under 40× power by Leica Aperio CS2 scanning system. Skeletal muscle image analysis was conducted using Aperio ImageScope. Briefly, 4 regions of interest (ROIs) were analyzed in each of the two muscles: Gastrocnemius and soleus. Quantification of muscle fibrosis was determined by the percentage of fibrotic (blue) tissue present within the section (7.5 mm^2^ tissue area) and by the average fiber cross‐sectional area within the ROI. Myofiber maturity was classified based on the position of the nuclei (peripheral nuclei indicating mature fibers; centralized nuclei indicating regenerating/immature fibers) using the same ROIs.

### 2.5 Poly RNA sequencing

#### 2.5.1 Library Construction and Sequencing

For RNAseq, 10 additional rats were randomly divided into IFCO (*n* = 5) and sham (*n* = 5) surgery groups. The gastrocnemius and soleus muscles were dissected from the rats 4 weeks post‐IFCO or sham surgery. A poly (A) RNA sequencing library was prepared following Illumina's TruSeq‐stranded‐mRNA sample preparation protocol. RNA integrity was checked with Agilent Technologies 2100 Bioanalyzer. Poly(A) tail‐containing mRNAs were purified using oligo‐(dT) magnetic beads with two rounds of purification. After purification, poly(A) RNA was fragmented using a divalent cation buffer at elevated temperatures. The DNA library construction is shown in the following workflow. Quality control analysis and quantification of the sequencing library were performed using Agilent Technologies 2100 Bioanalyzer High Sensitivity DNA Chip. Paired‐end sequencing was performed on Illumina's NovaSeq 6000 sequencing system.

#### 2.5.2 Bioinformatics Analysis

Cutadapt [37] and in‐house Perl scripts were used to remove reads containing adapter contamination, low‐quality bases, and undetermined bases, and sequence quality was verified using FastQC (v.0.10.1). The resulting clean reads were mapped to the *Rattus norvegicus* reference genome (Ensembl release 104) using the splice‐aware aligner HISAT2 [38]. The mapped reads of each sample were assembled using StringTie, and the per‐sample transcriptomes were then merged to reconstruct a comprehensive transcriptome using Perl scripts and gffcompare.

StringTie [39] was used to perform expression levels for mRNAs by calculating FPKM. mRNAs differential expression analysis was performed by the R package DESeq2 [40] between two different groups (and by the R package edgeR [41] between two samples). The mRNAs with the parameter of false discovery rate (FDR) below 0.05 and absolute fold change ≥ ±2 were considered differentially expressed mRNAs. Overrepresentation of Gene Ontology (GO) terms and Kyoto Encyclopedia of Genes and Genomes (KEGG) pathways among DEGs was evaluated by the hypergeometric test. Gene set Enrichment Analysis (GSEA) [42] was additionally performed on the ranked gene list against the GO and KEGG gene set collections using the Signal2Noise metric and 1 000 gene‐set permutations (gene‐set size 15–500). Gene sets and pathways were considered significantly enriched at a nominal *p*‐value < 0.01 and an FDR q‐value < 0.25. The threshold FDR < 0.25 is the cut‐off recommended by the developers of GSEA for exploratory analysis at the gene set level, where the conventional FDR < 0.05 is overly conservative and can miss the coordinated, modest‐magnitude changes GSEA is designed to detect [42]. Sequencing and bioinformatic analysis were completed by LC Sciences LLC, TX, USA.

#### 2.5.3 Validation

Differentially expressed genes thought to contribute to the muscle's observed histopathology were further validated by quantitative PCR (qPCR). qPCR was carried out with an Applied Biosystems StepOnePlus qPCR System using SYBR Green Master Mix (Roche, Indianapolis, IN, USA) and gene‐specific primers. The Ct (threshold cycle) values of the target gene amplifications were normalized to those of the actin beta (Actb). Each biological replicate was measured with three technical replicates, and fold change was calculated using the 2 − ΔΔCT method. Primers (Table 1) were designed and synthesized by Integrated DNA Technologies (Coralville, IA, USA) based on published sequences (http://www.ncbi.nlm.nih.gov).

**TABLE 1:** Oligonucleotide primers for qPCR.

Gene symbol	Forward (5′ – 3′)	Reverse (5′ – 3′)
COL1A1	ccaatggtgctcctggtatt	gttcaccactgttgcctttg
COL3A1	gtgtgatgatgagccactagac	tgacaggagcaggtgtagaa
COL5A1	aaggagaacagggcattacag	gacccgatgaacctttagca
COL8A1	gcaaagagtacccacacctac	ctcctcgcaaactggctaat
CASP8	tgtcctcgaggtgaggatatt	tgttcctcgggttgtctttatt
FGF10	caacggcaggcaaatgtatg	gaagtgagcggaggtgttt
FGFR1	cctgaagactgctggagttaat	tgatgggagagtccgatagag
FN1	ccaagtacattctcaggtggag	ggtcaggcctttgatggtatag
KLF5	ccatgccgagtcagtttctt	ggagcatctcagcttgtctatc
MYOF	ttgacctggtgattggctatac	cctggtcttcttcctcttcttc
PTGFRN	ccaaacctcaggtcccatattt	gtccacagtgacgatacagaac
TGFBI	ggctgcatcaggactcaata	ttcaaggtctcagcaggaatc
VEGFC	cccaacaaggagttggatga	cacactggcatgagtctctatc

### 2.6 Statistical Analysis

Statistical evaluation was analyzed using GraphPad Prism (GraphPad Software, San Diego, CA. Version 9.4.1). Normality was first determined using the Shapiro–Wilk test with an alpha set at 0.05. If data sets were not normally distributed, Mann–Whitney tests were utilized to determine differences between sham and IFCO groups. Mann–Whitney was utilized again when determining differences between IFCO soleus and IFCO gastroc. Differences between the two groups (Sham and IFCO) were defined at *p* < 0.05, being statistically significant. A priori power was calculated using an effect size ≥ 1, power = 0.80, allocation ratio = 0.5, and α = 0.05.

## 3 Results

### 3.1 Hindlimb Blood Flow Following IFCO

Immediately following IFCO, resting limb perfusion measured by LDPI was reduced by approximately 70% in rats when comparing the ipsilateral with the contralateral hind paws. Reduced perfusion was observed for up to 28 days. By day 42, resting hind‐paw blood flow was no longer significantly different between sham and IFCO rats (Figure 1C,D).

### 3.2 Muscle Structural Alterations After IFCO Introduction

**FIGURE 2:**
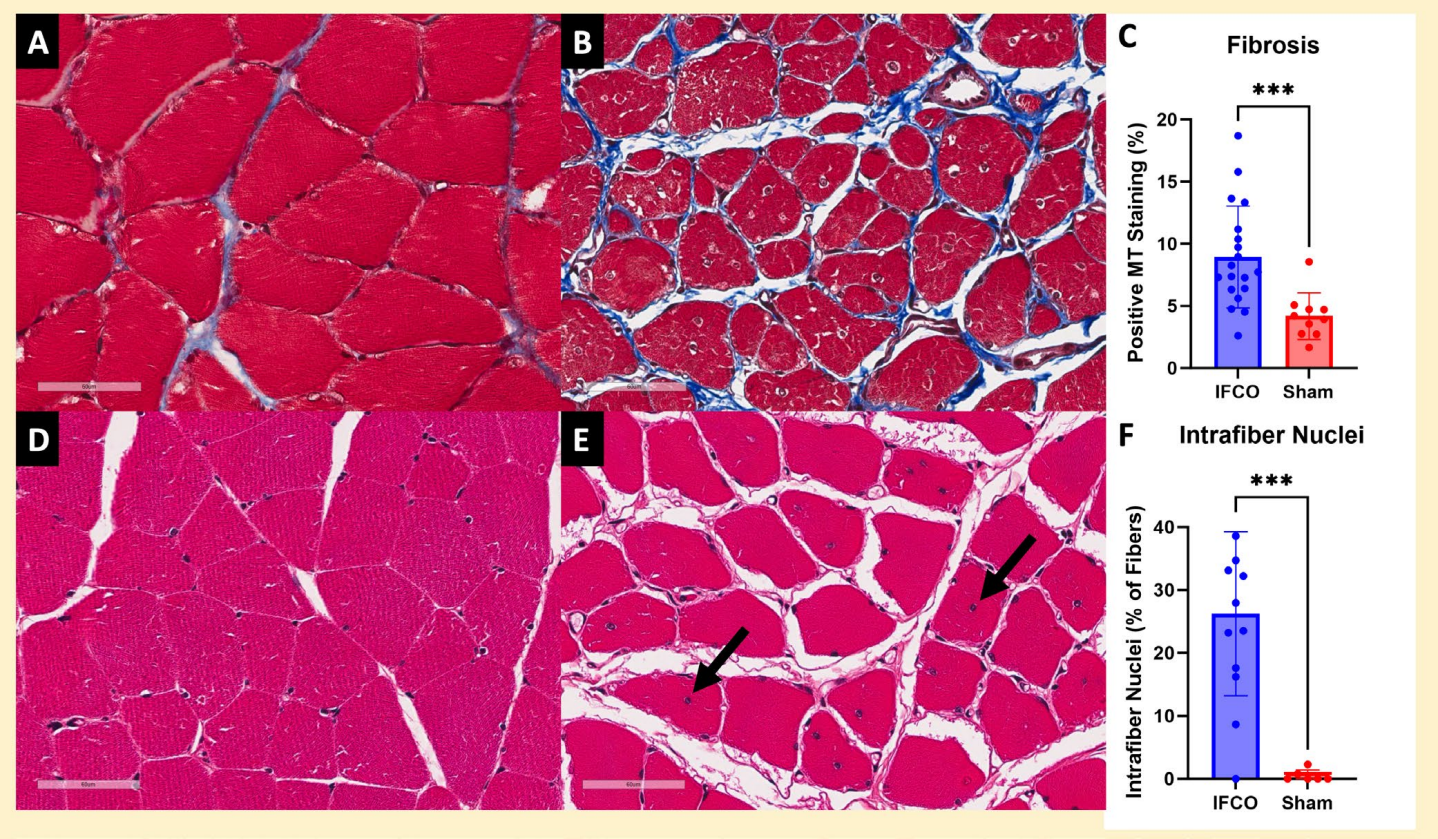
Histological characterization before and after IFCO. Masson's trichrome staining of sham (A) and IFCO (B) rat triceps surae muscles, with quantification of positive (blue) staining indicating fibrosis (C). Hematoxylin and eosin (H&E) staining of sham (D) and IFCO (E) muscles, with quantification of fibers exhibiting intrafiber/centralized nuclei pointed by arrows (F). All assessments were performed 4 weeks post‐surgery. Mean ± SD. *p* < 0.001 (***), *n = 11–19 per group*.

**FIGURE 3:**
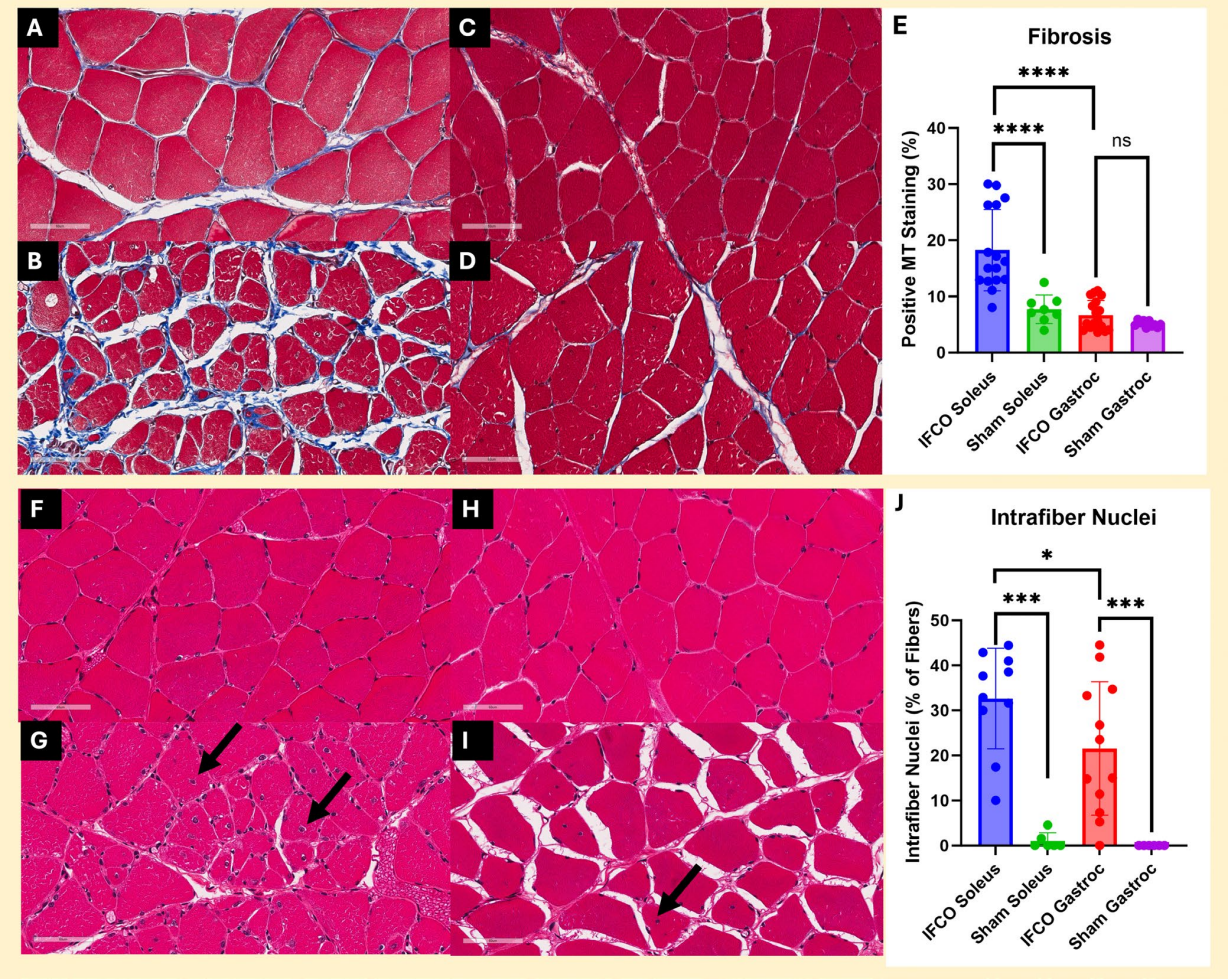
Histological analysis of the soleus and gastrocnemius muscles. Masson's trichrome staining of sham soleus (A), IFCO soleus (B), sham gastrocnemius (C), and IFCO gastrocnemius (D), with quantification of positive (blue) staining indicating fibrosis (E). Hematoxylin and eosin staining of sham soleus (F), IFCO soleus (G), sham gastrocnemius (H), and IFCO gastrocnemius (I), with quantification of fibers exhibiting intrafiber/centralized nuclei (arrows) (J). All measurements were performed 4 weeks post‐surgery. Mean ± SD. *p* < 0.05 (*), *p* < 0.001 (***), *p* < 0.0001 (****), *n = 11–19 per group*.

When the triceps surae muscle was analyzed as a whole, levels of fibrosis, as measured by positive MT staining, were significantly elevated (sham 4.19% vs. IFCO 8.94%, *p*‐value = 0.0005). Concurrently, H&E staining showed increased proportions of centralized nuclei (sham 0.51% vs. IFCO 26.23%, *p*‐value = 0.0008), indicating prior myofiber injury with regeneration (Figure 2A–F). Notable differences in fibrosis, centralized nuclei, and cross‐sectional area (CSA) could be observed between soleus and gastrocnemius (gastroc). This led us to investigate the muscles separately. In doing so, elevated levels of fibrosis were ascertained between IFCO and sham solei (sham 4.99% vs. IFCO 13.19%, *p*‐value < 0.0001), but no significant differences were seen between the IFCO and sham gastroc (sham 4.71% vs. IFCO 5.92%, *p*‐value = 0.3816). Furthermore, the IFCO solei exhibited higher levels of fibrosis when compared to the IFCO gastroc (soleus 13.19% vs. gastroc 5.92%, *p*‐value < 0.0001) (Figure 3A–E). Similar results were seen when evaluating centralized nuclei. Both IFCO muscles revealed increased proportions of centralized nuclei, but the soleus had significantly more than the gastroc (soleus 32.65% vs. gastroc 21.55%, *p*‐value = 0.0043). In contrast, the sham muscles had little to no centralized/intrafiber nuclei (Figure 3F–J).

**FIGURE 4:**
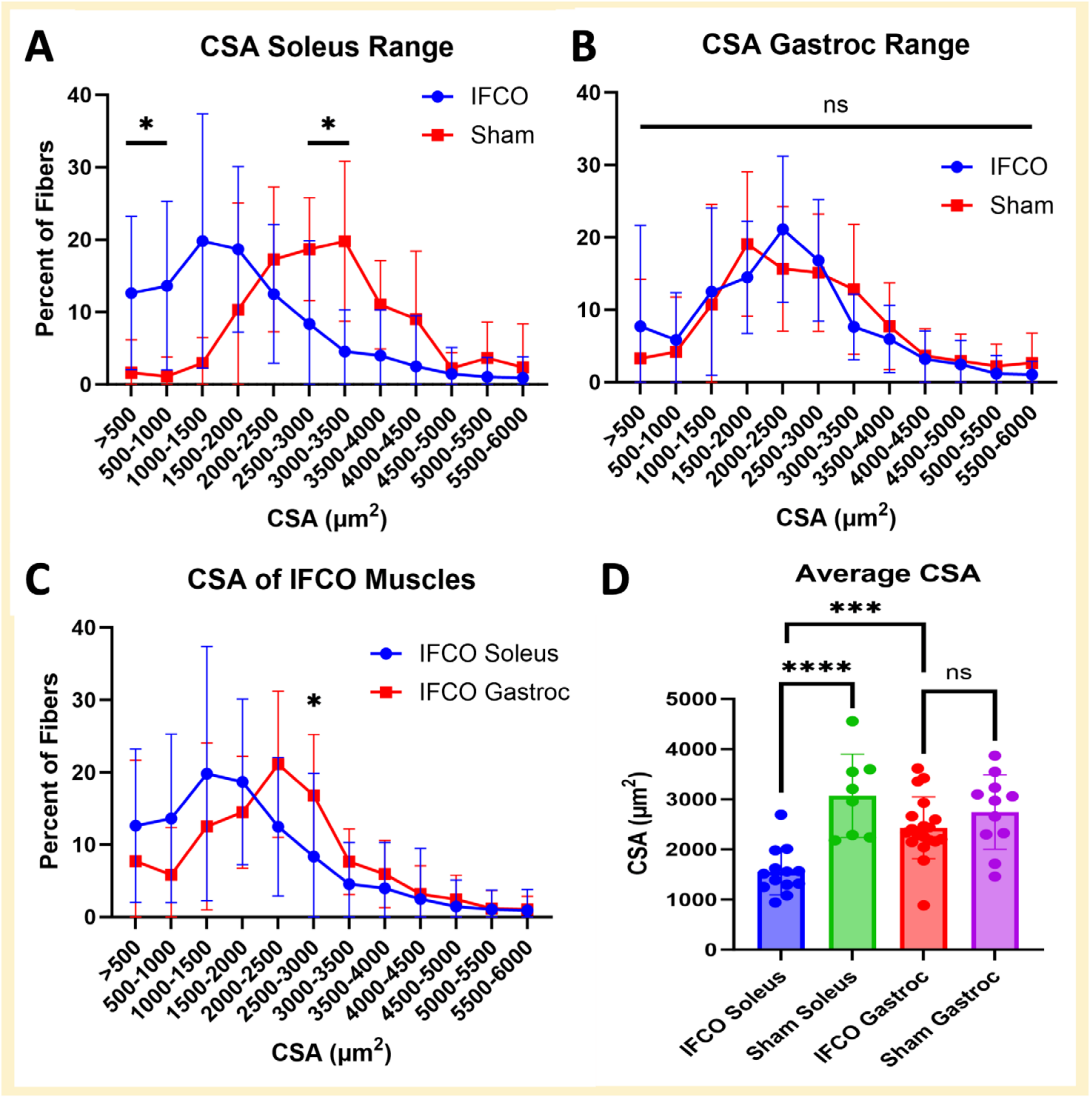
Cross‐sectional area of the soleus and gastrocnemius with or without IFCO. Distribution of muscle fiber cross‐sectional areas in the soleus (A) and gastrocnemius (B) following sham or IFCO surgery. Comparison of fiber‐area distributions between IFCO soleus and IFCO gastrocnemius (C). Mean cross‐sectional area of soleus and gastrocnemius fibers after sham or IFCO surgery (D). All measurements were obtained 4 weeks post‐surgery. Mean ± SD. *p* < 0.05 (*), *p* < 0.001 (***), *p* < 0.0001 (****), *n = 11–19 per group*.

Fiber morphology was also compromised in IFCO solei. Analysis of CSA stratifications showed that IFCO solei had higher proportions of small fibers (> 1 000 μm^2^) and reduced proportions of mid‐sized fibers (2 500–3 500 μm^2^) when compared to sham solei (Figure 4A). In contrast, the CSA distributions of gastroc muscles from IFCO rats did not differ from those of sham rats (Figure 4B). Moreover, when the IFCO muscles were compared against one another, the solei displayed trends of higher quantities of smaller fibers and reduced mid‐sized fibers, but only the percentage of fibers between 2 500–3 000 μm^2^ differed significantly (Figure 4C). These data were consistent when the average CSA was assessed. The average CSA was reduced from approximately 3 000 μm^2^ in sham solei to ~1 500 μm^2^ in IFCO solei (Figure 4D). Differences were also seen between IFCO solei and gastrocnemii, but not between IFCO and sham gastrocnemii.

### 3.3 Gene Expression Profiles Between Sham Soleus and Gastrocnemius

**FIGURE 5:**
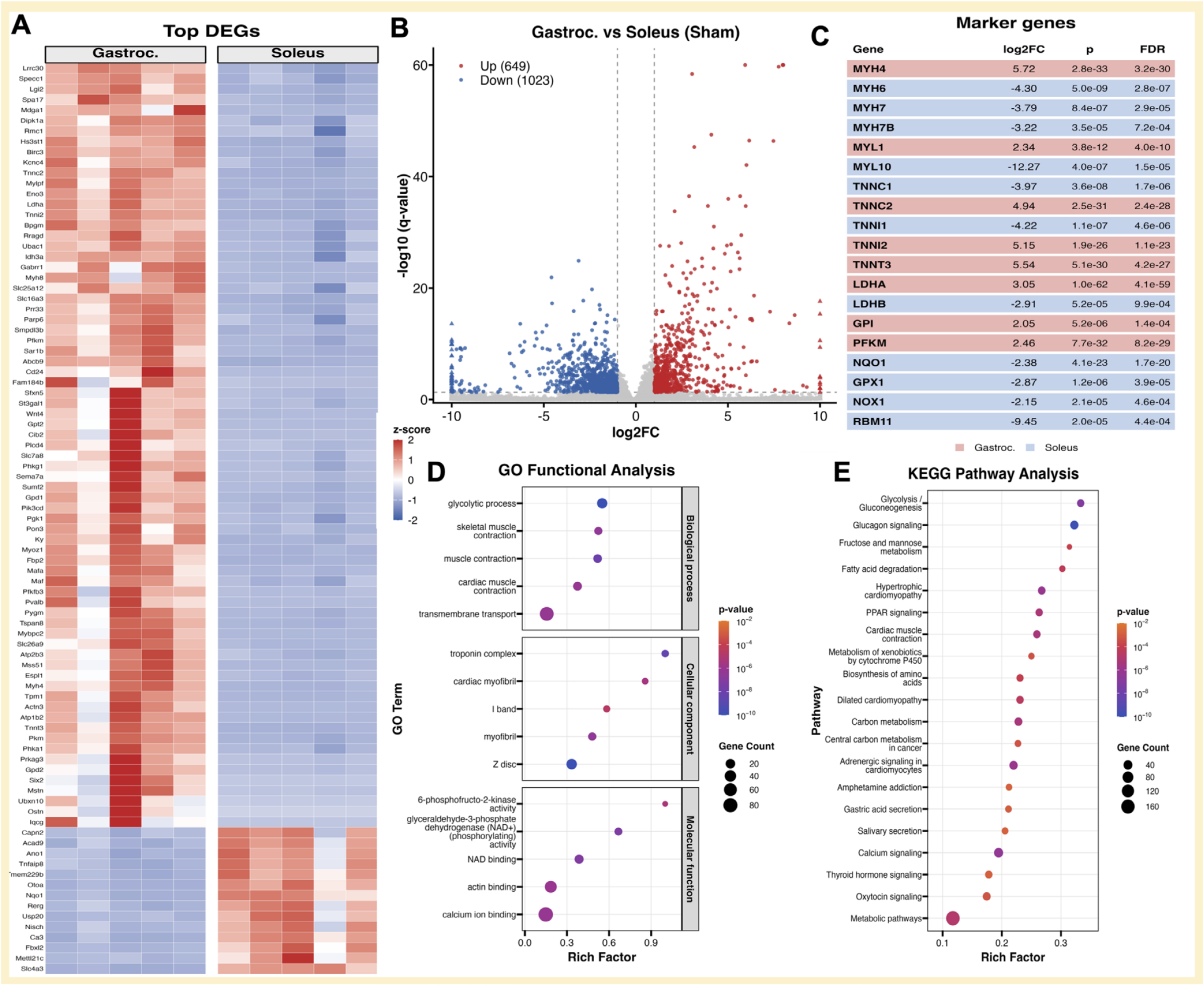
Baseline transcriptional differences between soleus and gastrocnemius muscles. Heatmap (A) and volcano plot (B) showing the top 100 differentially expressed genes between soleus and gastrocnemius muscles in healthy rats, with red indicating upregulation and blue indicating downregulation. (C) Differentially expressed genes (DEGs) between the two muscle types. (D, E) GO and KEGG pathway enrichment analyses. Rich factor (%) is the ratio of the number of differentially expressed genes annotated in a pathway (as indicated in the y‐axis) to the number of all genes annotated in this pathway. GO, Gene Ontology; KEGG, Kyoto Encyclopedia of Genes and Genomes.

Differences in muscle morphological outcomes suggested that ischemia had differentially impacted the muscles. We hypothesized that higher proportions of type I fibers would enhance muscle susceptibility to ischemic damage. As expected, the sham gastroc had a different transcriptomic profile compared to the sham soleus (Figure 5A,B). The gastroc possessed higher expression of myosin heavy chain 4 (MYH4) and troponin subsets (TNNC2, TNNI2, and TNNT3) and other gene markers for type II fibers. In contrast, the soleus highly expressed MYH7, TNNC1, TNNI1, TNNI3, and other type I fiber contractility genes (Figure 5C). These results indicate that the gastroc contained greater proportions of type II fibers, while the soleus predominantly comprises type I fibers. Gene set enrichment analysis further highlighted fiber composition differences by showing upregulation of glycolytic genes, such as glucose phosphate isomerase (GPI), Lactate dehydrogenase A (LDHA), and 6‐phosphofructokinase, muscle type (PFKM), within the gastroc, while the soleus upregulated Lactate dehydrogenase B (LDHB) and NADPH oxidase 1 (NOX1). The soleus also has unregulated NADPH dehydrogenase quinone 1 (NQO1), Glutathione peroxidase 1 (GPX1), and RNA Binding Motif Protein 11 (RBM11), consistent with other reports stating that type I fibers possess higher anti‐oxidative capacity (Figure 5C). In total, almost 1700 DEGs were identified between the sham muscles, with most DEGs being downregulated in the gastroc compared to the soleus.

### 3.4 Effects of IFCO on the Transcriptomes of the Soleus and Gastrocnemius Muscles

**FIGURE 6:**
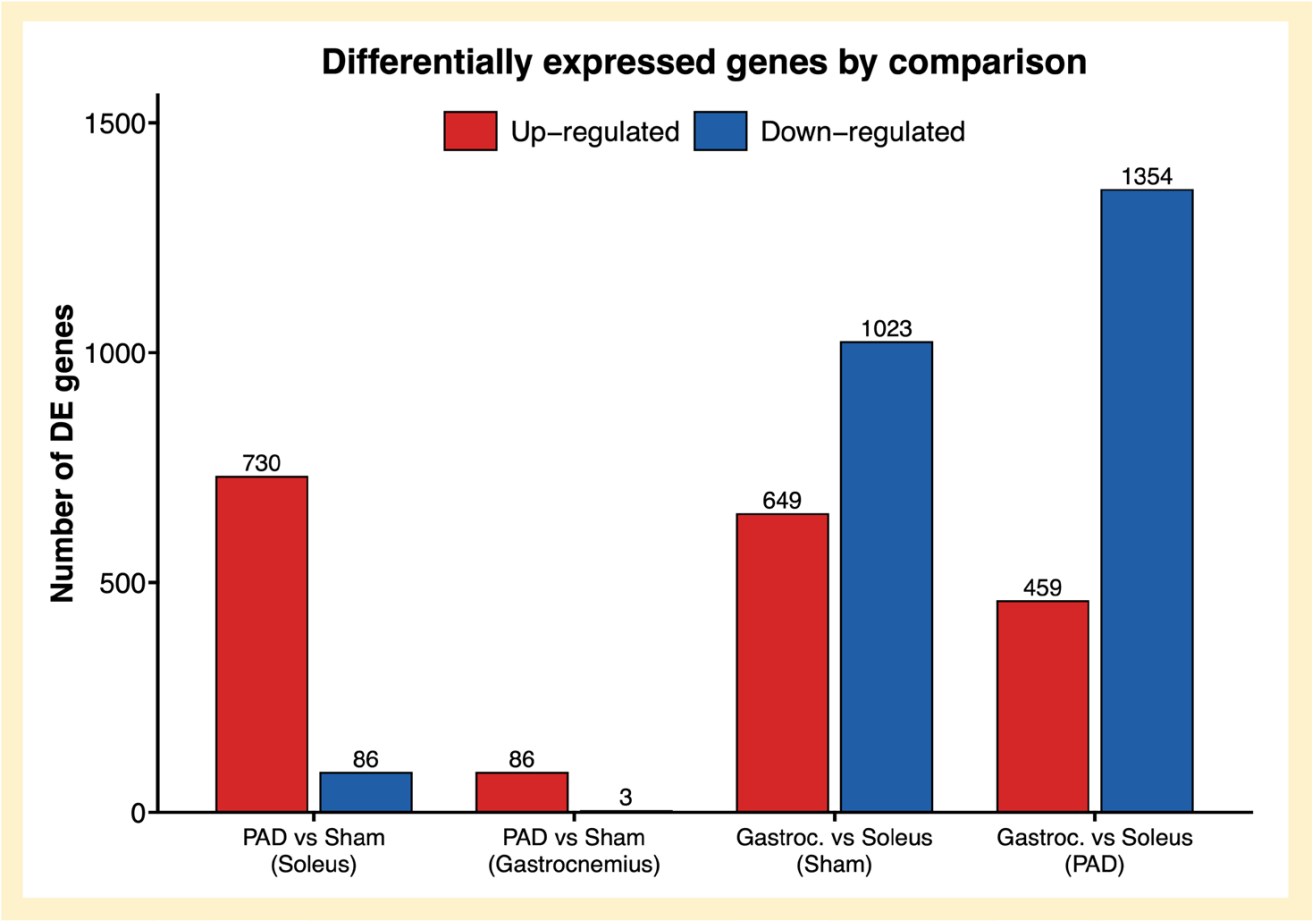
Bulk RNA‐seq analysis of gene expression profiles in the soleus and gastrocnemius muscles from sham and IFCO‐induced peripheral arterial disease (PAD) rats. Using the predefined differential expression criteria, 816 differentially expressed genes (DEGs) were identified in the soleus following IFCO, including 730 upregulated and 86 downregulated genes compared with sham controls. In the gastrocnemius, 89 DEGs were identified, of which 86 were upregulated and 3 were downregulated. Consistent with their distinct muscle fiber composition, the sham gastrocnemius and soleus exhibited markedly different transcriptomic profiles. Approximately 1 700 DEGs were identified between the sham gastrocnemius and soleus muscles, with the majority of DEGs showing lower expression in the gastrocnemius relative to the soleus. Similarly, more than 1 800 DEGs were identified between IFCO‐treated gastrocnemius and soleus muscles, with most DEGs remaining downregulated in the gastrocnemius.

Bulk RNAseq discovered that over 1800 DEGs were revealed when comparing IFCO soleus and gastroc with the most downregulated DEGs in the gastroc (Figure 6). A total of 816 DEGs were identified between IFCO and sham solei, with 730 upregulated and 86 downregulated genes. Conversely, only 89 DEGs were identified between the IFCO and sham gastrocs, with all but 3 being upregulated.

#### 3.4.1 IFCO Soleus Transcriptome Becomes Pro‐Fibrotic

**FIGURE 7:**
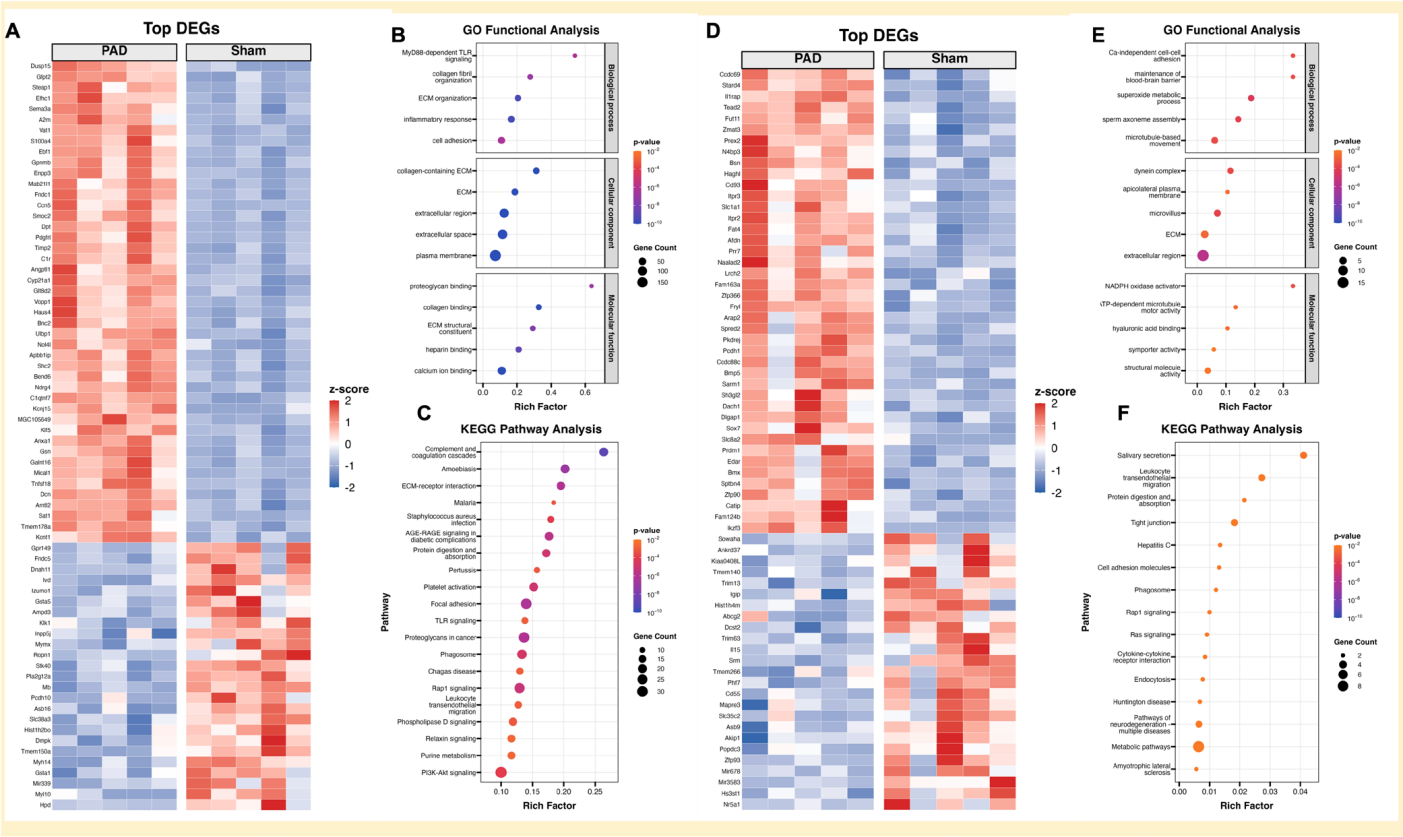
Bulk RNA‐seq analysis of the soleus (A*–*C) and gastrocnemius (D*–*F) after IFCO. (A) Heat map of the top 70 differentially expressed genes (DEGs) in the soleus, ranked by *p*‐value, with red indicating higher expression and blue indicating lower expression. (B) Top 20 enriched KEGG pathways and (C) top 15 enriched Gene Ontology (GO) terms identified from soleus DEGs, ranked by enrichment *p*‐value. (D) Heat map of the top 70 differentially expressed genes in the gastrocnemius, ranked by *p*‐value. (E) Top 20 enriched KEGG pathways and (F) top 15 enriched GO terms identified from gastrocnemius DEGs, ranked by enrichment *p*‐value. *n* = 5 per group. Rich factor (%) represents the ratio of the number of differentially expressed genes annotated to a given pathway to the total number of genes annotated to that pathway. KEGG, Kyoto Encyclopedia of Genes and Genomes; GO, Gene Ontology.

**FIGURE 8:**
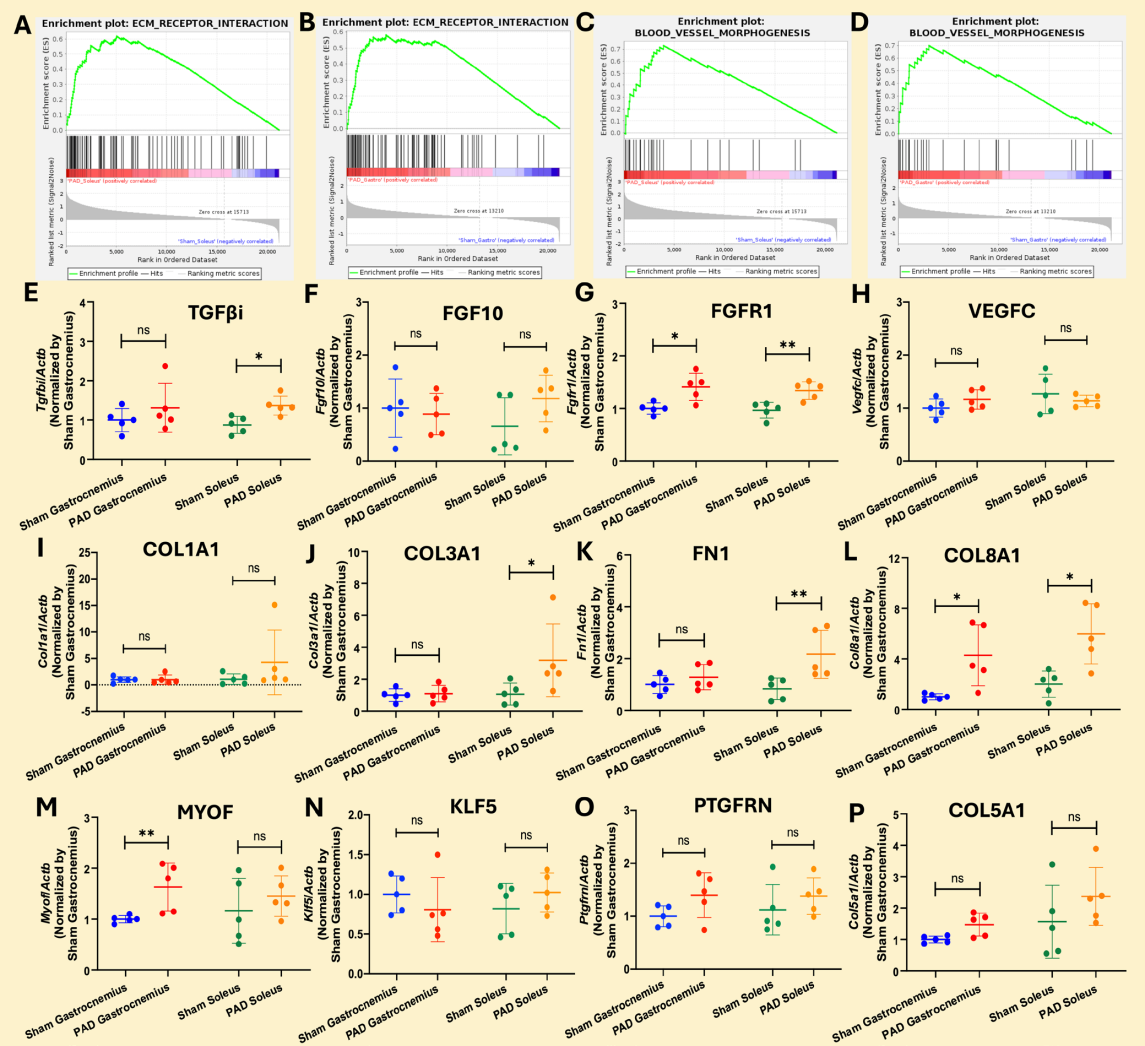
qPCR validation of bulk RNAseq findings. Enrichment plots showing ECM–receptor interaction in soleus (A) and gastrocnemius (B), and blood vessel morphogenesis in soleus (C) and gastrocnemius (D) following sham vs. IFCO surgery. (E–P) qPCR validation of select genes associated with myopathic alterations, including TGFβi, FGF10, FGFR1, VEGFC, COL#A1, FN1, MYOF, KLF5, and PTGFRN. TGFβi (transforming growth factor β induced), FGF10 (fibroblast growth factor 10), FGFR1 (fibroblast growth factor receptor 1), VEGFC (vascular endothelial growth factor type c), COL#A1 (collagen # type alpha 1), FN1 (fibronectin 1), MYOF (myoferlin), KLF5 (Kruppel‐like factor 5), PTGFRN (Prostaglandin F2 Receptor Negative Regulator). Mean ± SD. *n = 5 per group*. *p* < 0.05 (*) and *p* < 0.01 (**), ns—not significant.

Functional and gene set enrichment analyses were performed on differentially expressed genes (DEGs) (Figure 7A) to uncover unique transcriptional signatures of the soleus following IFCO or sham surgery. KEGG pathways and GO terms were ranked based on *p*‐value; some of the most enriched pathways consisted of “complement and coagulation cascades,” “focal adhesion,” and “ECM‐receptor interactions,” and the top 6 upregulated GO terms involved collagen and the extracellular matrix (Figure 7B,C). These results suggest that ECM production and its interactions with cells are enhanced (Figure 8A,B). Many growth factors reported to promote fibrosis were observed to be upregulated in the IFCO soleus, such as transforming growth factor‐beta inducible (TGFβi), fibroblast growth factor 10 (FGF10), insulin‐like growth factor 1 (IGF1), and early response growth factor 1 (EGR1), among others (Table 2). Such growth factors can stimulate signaling pathways Rap1 and PI3K‐Akt, which have been implicated in fibrosis [43, 44, 45] and found to be enriched after IFCO. Downstream impacts were also demonstrated by the heightened expression of platelet‐derived growth factor receptor alpha (PDGFRA), indicating increased fibroblast proliferation and greater Fibroblast activation protein‐α (FAP) and NADPH oxidase 4 (NOX4), evincing activation of fibroblasts. Activation of fibroblasts is substantiated by the increased expression of collagen subtypes, fibronectin (FN1), and other ECM components (Table 2). However, qPCR did not confirm the upregulation of some components and growth factors, such as COL1A1 (Figure 8I), FGF10 (Figure 8F), and vascular endothelial growth factor C (VEGFC) (Figure 8H).

**TABLE 2:** DEGs of the sham and IFCO soleus that contribute to the observed IFCO‐induced myopathy.

Gene name	log2 (fc)	NOM *p*‐value	FDR q‐value
COL1A1	2.02	0.00	0.00
COL3A1	2.31	0.00	0.00
COL4A1	1.08	0.00	0.04
COL5A1	1.83	0.00	0.00
COL6A1	1.67	0.00	0.00
COL8A1	1.84	0.00	0.00
EGFR	1.63	0.00	0.02
EGR2	3.73	0.00	0.00
EGR3	3.06	0.00	0.03
FAP	2.04	0.00	0.00
FBN1	1.47	0.00	0.00
FGF10	2.13	0.00	0.03
FGFR1	1.10	0.00	0.00
FN1	1.83	0.00	0.00
IGF1	1.49	0.00	0.00
LOX	1.99	0.00	0.00
LOXL2	2.05	0.00	0.03
NOX4	1.92	0.00	0.00
PDGFRA	1.51	0.00	0.00
TGFBI	1.32	0.00	0.02
VEGFC	1.05	0.00	0.01

*Note:* IFCO soleus vs. sham soleus; positive log2(fc) indicates that the gene is enhanced in the IFCO soleus compared to sham soleus.

#### 3.4.2 IFCO Soleus Transcriptome Becomes Angiogenic 4 Weeks After Surgery

Enrichment plots of the soleus show that the IFCO soleus undergoes enhanced blood vessel morphogenesis (Figure 8C,D). Notably, the upregulated collagen subtypes, COL3A1 and COL8A1, and FN1 (Figure 8J–L) are also angiogenic and related to tissue repair [46, 47, 48, 49, 50]. In addition, COL3A1 and COL8A1 are critical components of vessel adventitia; therefore, the recognized fibrotic response seen in the histological sections may be an aftereffect of an angiogenic reaction.

#### 3.4.3 Both IFCO Muscles Undergo Myogenesis and Regeneration

Increased proportions of centralized nuclei in the soleus and gastroc IFCO muscles suggest that they are undergoing regeneration. Comparing the IFCO soleus to its sham counterpart, GSEA identified elevated expression of myoblast fusion genes, like myoferlin (MYOF), prostaglandin F2 receptor negative regulator (PTGFRN), kruppel‐like factor 4 and 5 (KLF4 and 5), and cluster of differentiation 9 (CD9), corroborating with centralized nuclei seen in histological analysis. RNAseq also revealed that the IFCO soleus, compared to the gastroc, had significantly higher expression of satellite marker paired box protein 7 (PAX7), myogenic factor 5 (MYF5), and myogenin (MYOG), while the gastroc overexpressed myogenic determination 1 (MYOD1), myostatin (MSTN), and myozenin 1 and 3 (MYOZ1 and 3) (Table 3). However, qPCR found only MYOF to be significantly upregulated and only when comparing IFCO and sham gastrocs (Figure 8M). Antithetical data most likely arose from the differing methods. Similar discrepancies were observed again when analyzing apoptotic genes.

**TABLE 3:** IFCO soleus and gastroc DEGs that contribute to IFCO‐induced myogenesis.

Gene name	log2 (fc)	NOM *p*‐value	FDR q‐value
MSTN	3.43	0.00	0.00
MYF5	−1.98	0.00	0.00
MYOD1	2.42	0.00	0.00
MYOF	−1.28	0.00	0.02
MYOG	−1.96	0.00	0.00
MYOZ1	3.76	0.00	0.00
MYOZ3	1.29	0.00	0.00
PAX7	−1.05	0.00	0.01

*Note:* IFCO soleus vs. IFCO gastroc; positive log2(fc) indicates that the gene is enhanced in the IFCO soleus compared to IFCO gastroc.

#### 3.4.4 IFCO Soleus Transcriptome Is Apoptotic, but Muscle Cells Do Not Exhibit an Apoptotic Phenotype

**FIGURE 9:**
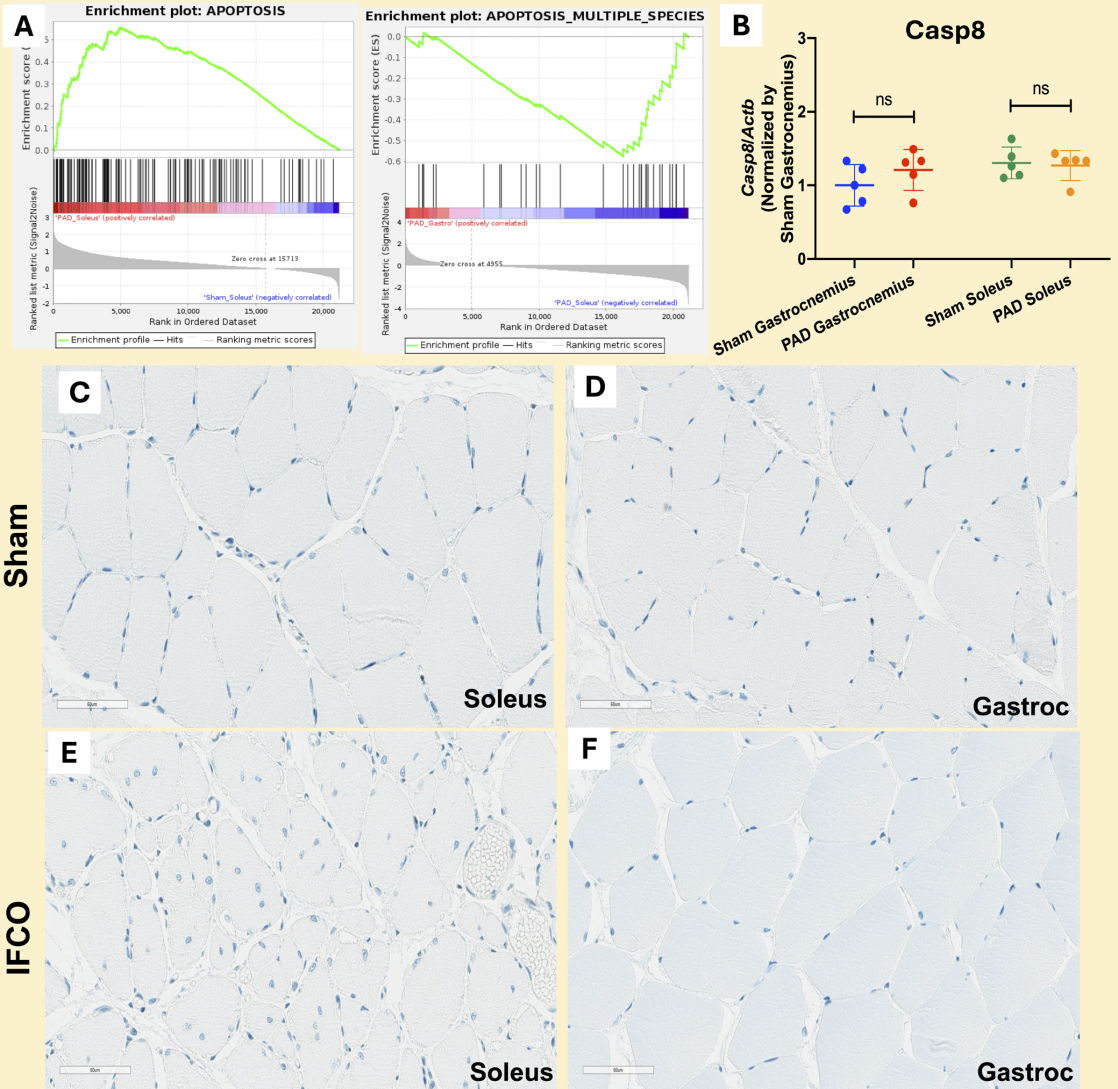
Apoptotic fiber response following IFCO. (A) Enrichment plots of apoptosis‐related pathways in soleus (left: IFCO vs. sham) and in gastrocnemius (right: IFCO gastrocnemius vs. IFCO soleus). (B) qPCR analysis of caspase‐8 expression in sham vs. IFCO soleus and gastrocnemius. (C–F) Anti‐CC3 immunohistochemistry in sham soleus (C), IFCO soleus (E), sham gastrocnemius (D), and IFCO gastrocnemius (F). Mean ± SD. *n = 5 per group*. ns—not significant.

With the increase of pro‐survival genes, it was surprising to see increased caspase 3 (Casp3) and Casp8 expression. However, qPCR failed to confirm heightened expression of Casp8, and IHC did not display any evidence of cleaved caspase 3 (CC3), meaning that muscle fibers do not undergo apoptosis 4 weeks after IFCO (Figure 9).

#### 3.4.5 IFCO Gastroc Transcriptome Is Largely Unchanged After 4 Weeks

When comparing the IFCO and sham gastrocs, only 89 DEGs were identified. Based on the cut‐off criteria, KEGG identified 4 enriched pathways consisting of genes related to “salivary secretion,” “leukocyte trans‐endothelial migration,” “lipoic acid metabolism,” and “tight junction” (Figure 7F). Based on histological data showing no fibrosis, it was consternation that the most significantly enriched GO term—regarding *p*‐value and the number of genes—involved the “extracellular region” (Figure 7E). Furthermore, qPCR demonstrated that FGFR1 (Figure 8G) and COL8A1 (Figure 8L) were significantly upregulated in the IFCO soleus and that COL5A1 (Figure 8P) approached significance. Like the soleus, the IFCO gastroc was anti‐apoptotic; neither Casp3 nor Casp8 was upregulated, as confirmed by qPCR and IHC anti‐CC3 staining (Figure 9). Unlike the soleus, the IFCO gastroc upregulated the expression of MYOF, as measured by qPCR (Figure 8M).

## 4 Discussion

In this study, we examined the use of a new ilio‐femoral endovascular catheter occlusion HLI model in rats. Preclinical animal models are essential for developing novel therapies, but no therapeutic approach for PAD has been successfully translated to human trials. Current models of HLI do not fully recapitulate human disease; therefore, outcomes are much more difficult to extrapolate to humans. Our findings demonstrate that IFCO induces sustained reductions in blood flow, skeletal muscle myopathy, and transcriptomic alterations. Most importantly, IFCO‐induced myopathy and transcriptomic changes differed between the soleus and gastrocnemius muscles.

At the cellular level, PAD patients develop an ischemic myopathy in their leg muscles characterized by myofiber degeneration (centralized nuclei, loss of standard polygonal shape, and lower cross‐sectional areas) and increased fibrosis [51, 52, 53, 54, 55]. Our rat data closely align with these observations, but IFCO in rats caused disproportionate damage to the soleus rather than the gastroc, which we presume is due to the predominance of type I fibers in the soleus [56, 57, 58, 59]. RNAseq data attested that the gastroc harbored a greater proportion of type II fibers, given the increased expression of MYH4, TNNC2, TNNI2, and TNNT3. Simultaneously, the soleus highly expressed MYH7 and other type I fiber contractility genes. The soleus also highly expressed NQO1, GPX1, and RBM11, consistent with other reports stating that type I fibers possess higher anti‐oxidative capacity [60, 61, 62], which could explain why type II fibers display greater levels of oxidative stress during PAD [53]. Of note, the soleus continued expressing more anti‐oxidative transcripts than the gastroc during IFCO conditions.

Consistent with the histological findings, RNAseq revealed that the IFCO soleus highly expressed several collagen subtypes compared to sham; however, qPCR confirmed only COL3A1 and COL8A1. Taken in context, these data may add to a more singular description: Angiogenesis. Several forms of angiopoietins are upregulated in the IFCO soleus. COL3A1 and COL8A1 are crucial aspects of tunica adventitia and basement membranes of endothelial cells [46, 47, 48, 49]. So, increased capillary beds and their supportive matrix could manifest as increased fibrosis in our histological evaluation.

Muscle regeneration occurs through a process known as myogenesis. Muscle stem cells, known as satellite cells, are ordinarily quiescent but are activated upon injury. Once activated, satellite cell populations will expand into myoblasts, which will differentiate and fuse to generate new muscle fibers or undergo self‐renewal and revert back to a quiescent state to replenish the stem cell pool [63, 64]. Quiescent satellite cells are recognized by the expression of PAX7 [65, 66]. Muscular trauma or disease induces the expression of muscle regulatory factors (MRFs), like MYOD1 and MYF5, which then mediate the activation of satellite cells. Activated satellite cells can thus be distinguished by the expression of PAX7, MYOD1, and MYF5 [67, 68, 69]. Kruppel‐like factors, such as KLF4 and 5, can enhance MRF initiation and thus contribute to satellite cell activation [70, 71, 72]. Downregulation of PAX7 is a feature of committed myoblasts, whereas cells that downregulate MYOD1 and MYF5 undergo self‐renewing proliferation. Expression of MYOG indicates early differentiation of myoblasts, which is followed by fusion [73]. RNAseq revealed that the IFCO soleus compared to the gastroc had significantly higher expression of PAX7, MYF5, and MYOG, while the gastroc overexpressed MYOD1, MSTN, and MYOZ1 and 3. Upregulation of these myogenic genes indicates that the soleus and gastroc were injured by IFCO‐induced ischemia and are in different states of recovery. Particularly, the high expression of PAX7 and MYF5 in the soleus implies that satellite cells are active and proliferating. Simultaneously, the increased transcription of MYOG suggests that myoblasts are differentiating and fusing, which would explain the increased proportions of centralized nuclei. In the gastroc, MSTN and MYOZ1 and 3 indicate that the muscle fibers are more mature. Myostatin is thought to control muscle hypertrophy through limiting fiber size and promoting quiescence of satellite cells [74, 75]. Myozenin is expressed explicitly in striated muscle and has been shown to correlate with muscle fiber maturation [76]. The gastroc also significantly expressed MYOD1 and MYOF, as evidenced by GSEA and qPCR, respectively. MYOF is indispensable for normal myoblast fusion [77, 78]. Taken all together, including its larger cross‐sectional areas of muscle fibers, the gastroc still contains significant populations of differentiating and fusing myoblasts but may be further along in its regeneration. Whether its hastened regeneration results from its fiber composition requires further study.

There is contention within the literature on which fiber type is most impacted. Some studies show atrophy predominantly in type II fibers [52, 53, 79], while others show it in type I fibers [54, 80, 81]. Another study applying snRNAseq revealed that patients with PAD displayed a relative increase in the proportion of type II myonuclei and a decrease in type I myonuclei compared with controls [82]. Importantly, all these studies only analyzed the gastroc, in which we observed considerably less damage. For animal models, analysis of the rat soleus better correlates with human responses. In addition, muscles primarily of one fiber type may support more rigorous conclusions. Emphasis away from the gastroc may be beneficial because it exhibits extreme variability in fiber type composition [55].

The severity of PAD is most commonly stratified using the Rutherford classification system into seven categories based on the severity of symptoms: 0: asymptomatic, 1: mild claudication, 2: moderate claudication, 3: severe claudication, 4: ischemic resting pain, 5: minor tissue loss, 6: major tissue loss [83]. Quantitative measurements like walking performance and ABI can be used to further discriminate between the categories and provide additional rigor [83, 84]. Varying grades of severity add to the complexity of developing an animal model because most models only encompass one level of severity. As most patients with PAD remain at or below category 4, we set out to produce an animal model of HLI without gangrene or necrosis.

Currently, the most widely used technique to induce animal HLI involves surgical ligation of the branches of the femoral artery followed by its excision, as described by Pu et al. [34]. This model became the mainstay of PAD animal studies because it caused chronic HLI without gangrene. Researchers have produced gangrene models to mimic CLTI [85, 86], but the models do not closely reflect its pathophysiology or clinical course [87, 88]. The femoral excision model has its own drawbacks: It is highly invasive, technically demanding, does not account for animal‐to‐animal anatomical variation, and does not consistently occlude collateral vessels. In addition, the conventional HLI model is an acute form of ischemia and may not recapitulate slow, progressive disease pathophysiology.

Ligating the femoral artery and its inflow and outflow arteries demands extensive dissection, with attendant iatrogenic damage to the surrounding tissues, including veins, nerves, and muscles, all of which are central to the recovery and the physiology of the ischemic hindlimb. This is especially true if all inflow and outflow arteries are to be identified and controlled. Skillful dissection can reduce the damage, but becoming technically adept takes time and experience. Even with meticulous surgical technique, the deep arteries of the thigh, such as the deep femoral artery (DFA), can only be found if muscle tissue is divided or removed. Anatomical variation further complicates reliable branch identification and control, and the creation of reproducible ischemia due to uninterrupted collateral flow. IFCO circumvents these limitations by (1) requiring isolation only of the distal segment of the femoral artery, (2) using an intra‐arterial catheter to obviate precise identification and ligation of all femoral branches, and (3) extending the catheter through the femoral and external and common iliac arteries to the aortic bifurcation thus occluding an arterial segment (common and external iliac arteries) that would otherwise require open pelvic exposure, reducing operative morbidity and accommodating collateral anatomic variability.

Arteriogenesis, caused by persistent obstruction, pertains to increases in the diameter and thickness of collateral vessels, resulting in substantial increases in blood flow through these vessels. The caudal gluteal, DFA, and iliacofemoral arteries are major sources of collateral flow in relation to the femoral artery, and the origin of these arteries varies significantly. One report found that only 2 out of 11 mice had their DFA branch off the femoral artery [89]. This is alarming because many groups use the DFA as a landmark for their mouse models and often confuse the proximal caudal femoral artery with the DFA [90, 91, 92, 93]. Figure 1 compiles observed collateral sources [85, 94] with the most common anatomical variations seen in BALB/c mice [89]. Although perfusion recovery differs between BALB/c and C57BL/6J mice strains [95, 96], no consistent interstrain differences in arterial anatomy have been demonstrated [97, 98]. However, unlike mice, rats do not possess DFAs but still possess abundant collateral networks at baseline [99], which could cause faster recovery of limb perfusion in rats after IFCO. Collateral vessels almost always branch off the internal or external iliac arteries above the inguinal ligament, despite their anatomical variation. Because most groups perform femoral excision distal to the inguinal ligament [100, 101, 102, 103, 104, 105, 106], collateral inflow vessels often remain patent. A recent report found high rates of inconsistent muscle damage in mice after the traditional femoral excision model [107]. The authors do not discuss ligating the DFA, but the thigh's minor and inconsistent muscle damage suggests that the DFA and other collateral vessels were not impacted by their surgical approach. The IFCO approach can mitigate collateral flow without dissecting above the inguinal ligament or exploring the entire femoral artery. To our knowledge, IFCO is the only Sprague Dawley rat HLI model that reliably reduces hindlimb blood flow for up to 4 weeks without genetic, dietary, or environmental manipulation, likely owing to enhanced hindrance of collateral perfusion.

Although our model improves upon the disadvantages of previous models, it utilizes an acute obstruction approach that changes the shear stress of collaterals, thereby promoting their growth into potentially adequate streams of flow. Krishna et al. referred to these procedures as 1‐stage HLI models and went on to show that using ameroid constrictors followed by ligation 2 weeks later (deemed 2‐stage HLI model) may better recapitulate human pathology because it does not lead to rapid alterations of shear stress and collateral development [106]. Even though IFCO can block most collaterals, one report showed that bilateral arterial ligation delayed blood flow recovery, suggesting that the collateral flow from the contralateral iliac artery plays a role in compensating for ischemia [85]. Still, IFCO provides an excellent framework for iterative HLI model design to achieve desired symptoms and phenotypes. It can be paired with ameroid constrictors for a more progressive occlusion and with transgenic mouse lines to study the impact of specific genes. The catheter is also scalable, meaning an investigator could insert a longer one that can extend into the aorta if desired. Lastly, IFCO models do not impede the integration of human comorbidities like smoking or diabetes. Overall, IFCO provides a robust foundation for the continued refinement of HLI models.

There are some limitations to this study that are worth noting. First, validation of our PAD model primarily focused on blood flow recovery, skeletal muscle histopathology, and gene expression profiles, with limited assessment of functional outcomes. In patients with PAD, the disease is associated with impaired walking capacity, altered gait patterns, and reduced overall locomotor activity [108]. Therefore, the extent to which our IFCO model recapitulates these clinically relevant functional deficits remains to be determined and warrants further investigation. Second, to facilitate simplicity of discussion, fiber type was referenced as the sole difference between the gastroc and soleus; however, the gastroc does contain type I fibers. Single‐nuclear RNAseq would improve the credibility of our conclusions by affording insight into fiber‐specific transcriptomes rather than muscle‐specific. Third, muscle histology and RNAseq were performed 4 weeks after IFCO. Because rats recover spontaneously, myopathy and transcriptomes likely differ at distinct time points. Fourth, muscle sections were performed halfway between the muscles' origin and the musculotendinous junction. Considering the complex patterns of perfusion that occur over the length of a muscle, it is reasonable that a diverse range of pathologies, which are not fully represented by the current analysis, occur based on the proximity of the myofibers to the site of artery blockage. A stereological analysis throughout the muscle would better describe the extent of muscle pathology. Fifth, although many genes were validated by qPCR, no protein measurements were performed. This is a considerable limitation given that skeletal muscle protein expression is weakly related to mRNA level [109]. Finally, all experiments were conducted on male rodents. Given the sex disparities of PAD, future studies are required to determine whether sex differences influence responses to ischemia.

## 5 Conclusion

Creating an animal HLI model that faithfully mirrors PAD is more complex than previously appreciated. The IFCO approach is technically straightforward, less invasive, and accommodates varied animal anatomy. In rats, IFCO induces sustained reductions in blood flow, with muscle‐specific myopathy and transcriptomic alterations without gangrene. To our knowledge, IFCO is the first Sprague Dawley rat HLI model to reduce hindlimb blood flow for up to 4 weeks in otherwise unmanipulated animals. IFCO without additional co‐morbidities produces muscle fibrosis and reduced cross‐sectional area, demonstrating that ischemia alone can drive myopathic alterations. Considering the pattern of chronic hypoperfusion, myopathy, and transcriptomic response, IFCO, as described, should be applied for the study of moderate PAD.

## Author Contributions

**Zhiqiu Xia:** methodology, validation, writing – review and editing, data curation. **Samuel Gillman:** methodology, validation, writing – review and editing, data curation, visualization. **Iraklis I. Pipinos:** methodology, conceptualization, visualization, investigation, writing – review and editing. **Oliver Kitzerow:** conceptualization, methodology, data curation, validation, investigation, writing – original draft, writing – review and editing. **Han‐Jun Wang:** conceptualization, investigation, methodology, validation, data curation, supervision, resources, project administration, visualization, funding acquisition, writing – original draft, writing – review and editing.

## Funding

This study was supported by NIH grant R01 HL171602‐01 and in part, by NIH grants R01 1R01HL172029‐01A1, HL‐152160‐01, HL‐169205‐01, and R21 HL170127‐01. Dr. Hanjun Wang is also supported by the Margaret R. Larson Professorship in Anesthesiology.

## Conflicts of Interest

The authors declare no conflicts of interest.

## Data Availability

The data that support the findings of this study are openly available in Figshare at https://figshare.com/account/items/30068758/edit., reference number 30068758.
